# Sex-Specific Associations of Monocyte-Based Inflammatory Indices with Renal Dysfunction and Hyperuricemia in the Saudi General Adult Population

**DOI:** 10.3390/healthcare14152374

**Published:** 2026-08-03

**Authors:** Hamood AlSudais, Dalal Alsowaida, Rehab B. Albakr, Abdulaziz S. Alsahli, Abdullah Alghamdi, Yazeed Alshuweishi, Abdulaziz M. Almuqrin

**Affiliations:** 1 Department of Clinical Laboratory Sciences, College of Applied Medical Sciences, King Saud University, Riyadh 12372, Saudi Arabia; 2 Department of Medicine, Division of Nephrology, College of Medicine, King Saud University, Riyadh 11472, Saudi Arabia

**Keywords:** CKD, eGFR, hyperuricemia, monocyte-to-high-density lipoprotein cholesterol ratio (MHR), monocyte-to-lymphocyte ratio (MLR), monocyte-to-neutrophil ratio (MNR), inflammation, inflammatory markers

## Abstract

**Background/Objectives:** Chronic kidney disease (CKD) is a major global health burden and is closely linked to persistent low-grade inflammation. Monocyte-based inflammatory indices, including the monocyte-to-high-density lipoprotein cholesterol ratio (MHR), monocyte-to-lymphocyte ratio (MLR), and monocyte-to-neutrophil ratio (MNR), have been associated with various metabolic and inflammatory disorders; however, their relationships with renal dysfunction and hyperuricemia remain insufficiently explored. This study examined the associations of these indices with renal dysfunction and hyperuricemia in the Saudi general population, with particular emphasis on sex-specific differences. **Materials and Methods:** This retrospective cross-sectional study included 9657 Saudi adults. Estimated glomerular filtration rate (eGFR) was calculated using the 2021 CKD-EPI creatinine equation. Associations of MHR, MLR, and MNR with reduced eGFR and hyperuricemia were examined using sex-stratified comparisons, correlation, risk assessment, receiver operating characteristic analysis, and regression modeling. **Results:** MHR was the strongest and most consistent marker associated with renal dysfunction. In females, reduced eGFR was associated with higher MHR and MLR, whereas in males it was associated with lower MHR, MLR, and MNR, indicating clear sex-specific divergence. Elevated MHR was associated with higher odds of reduced eGFR in females but lower odds in males. MHR also showed the strongest association with hyperuricemia in both sexes and remained independently inversely associated with eGFR. **Conclusions:** MHR showed the most consistent overall association with renal dysfunction and hyperuricemia among the evaluated indices. These findings suggest that MHR may provide a simple and accessible marker for sex-informed renal and metabolic risk assessment in the general population.

## 1. Introduction

Chronic kidney disease (CKD) is a progressive disorder characterized by persistent abnormalities in kidney structure or function [1]. It represents a major global health challenge, with an increasing burden reported in the Middle Eastern region [2,3]. In Saudi Arabia, recent population-based epidemiological data from a nationwide diagnostic laboratory network across all 13 administrative regions reported an overall CKD prevalence of 4.76% [4]. The study also showed a higher prevalence among males than females and a marked age-related increase in CKD prevalence, with increasing age, male sex, geographic region, and elevated hemoglobin A1c identified as significant predictors of CKD. In addition, recent community-based screening data from Riyadh further support this burden, showing that among 215 screened Saudi adults, 38% had abnormal eGFR values below 90 mL/min/1.73 m^2^, including 4% with eGFR below 60 mL/min/1.73 m^2^ and 34% with eGFR between 60 and 90 mL/min/1.73 m^2^ [5]. Lower eGFR was associated with older age, overweight/obesity, hypertension, and diabetes, highlighting the importance of early detection strategies in the Saudi population. CKD arises from diverse etiologies, including primary renal diseases such as glomerulopathies and secondary complications related to systemic conditions [6]. Early identification of individuals at risk of renal function decline is therefore essential for improving clinical outcomes and reducing disease burden [7].

Despite shared genetic backgrounds, men and women exhibit important biological differences that influence the risk of kidney disease. These differences may reflect the combined effects of sex chromosomes, sex hormones, kidney structure, renal hemodynamics, and immune-inflammatory regulation [8]. In particular, estradiol has been suggested to exert renoprotective effects through favorable effects on vascular tone, renal hemodynamics, inflammation, and fibrosis, whereas testosterone may contribute to glomerular hyperfiltration, vasoconstrictive responses, and profibrotic pathways [8]. These mechanisms may partly explain why men often experience faster kidney function decline and a higher risk of kidney failure, while the relative protection observed in women may change with age and hormonal status. Sex-related differences have been documented in CKD prevalence, progression, and outcomes [9,10].

Kidney disease and chronic low-grade inflammation are closely interconnected, with inflammation contributing to renal injury and becoming further amplified as kidney function declines, thereby promoting disease progression and related complications [11]. Immune cells are actively involved in this process, and alterations in circulating leukocyte populations may reflect the inflammatory burden associated with CKD [12,13].

In recent years, several inflammatory indices derived from routine complete blood count parameters have been investigated as simple and accessible markers for predicting and monitoring various disease states. Among these, monocyte-based indices, including the monocyte-to-HDL cholesterol ratio (MHR), monocyte-to-lymphocyte ratio (MLR), and monocyte-to-neutrophil ratio (MNR), have attracted increasing attention because monocytes are key mediators of innate immune activation and chronic inflammation. These indices are derived from readily available laboratory parameters and have been explored in a range of conditions, including cardiovascular disease, hepatic steatosis, type 2 diabetes, and anemia [14,15,16,17,18,19,20]. Given the growing burden of CKD and the need for accessible screening and monitoring tools, particularly in community and routine laboratory settings, these indices may provide practical value as simple inflammatory markers.

Although monocyte-based inflammatory markers have shown promise in several health disorders, their relationship with kidney function has not been comprehensively evaluated. In addition, the potential impact of biological sex on these associations remains insufficiently explored. Therefore, the present study comprehensively investigated the association of MHR, MLR, and MNR with kidney function and CKD risk in a large general Middle Eastern population, with particular emphasis on sex-specific differences.

## 2. Materials and Methods

### 2.1. Data Collection

This retrospective study was conducted following obtaining ethical approval from the Ethics Committee of King Saud University (Approval No. E-25-9893). Demographic and laboratory data from 9921 adults representing the general Saudi population who visited ELTA Laboratories between January 2022 and December 2022 were obtained from the ELTA Laboratories database, a large College of American Pathologists (CAP)-accredited diagnostic network primarily providing routine health screening services rather than specialized clinical care. Individuals younger than 18, missing creatinine measures, or with eGFR below 15 mL/min/1.73 m^2^ were excluded, as they would be on kidney replacement therapy [21]. Additional exclusion criteria, such as comorbidities or medication history, were not applied due to the nature of the data extracted from a general health testing institution. Considering the regulatory and cultural environment in Saudi Arabia, in which alcohol consumption is prohibited and rare, alcohol-associated effects on kidney function were not expected to influence the study population.

Monocyte-based inflammatory indices were manually calculated from absolute leukocyte counts and serum lipid measurements. MHR was determined as monocytes (×10^3^ cells/µL) ÷ HDL-C (mg/dL). MLR was calculated as monocytes (×10^3^ cells/µL) ÷ lymphocytes (×10^3^ cells/µL), and MNR was calculated as monocytes (×10^3^ cells/µL) divided by neutrophils (×10^3^ cells/µL). Optimal cutoff values for elevated indices were determined using receiver operating characteristic (ROC) curve analysis, with MHR > 0.00925, MLR > 0.188, and MNR > 0.174 considered high. Elevated UA values for males and females were defined according to the sex-specific reference ranges reported by Borai et al. [22]. eGFR was determined for the study population using the 2021 Chronic Kidney Disease Epidemiology Collaboration (CKD-EPI) creatinine equation [23]. For males, eGFR was calculated as: eGFR = 142 × min(SCr/0.9,1) ^−0.302^ × max(SCr/0.9,1)^−1.200^ × 0.9938^Age^, and for females as: eGFR = 142 × min(SCr/0.7,1) ^−0.241^ × max(SCr/0.7,1)^−1.200^ × 0.9938^Age^ × 1.012 [23]. Participants were categorized according to KDIGO GFR categories as follows: G1, eGFR ≥ 90 mL/min/1.73 m^2^; G2, eGFR 60–89 mL/min/1.73 m^2^; G3a, eGFR 45–59 mL/min/1.73 m^2^; G3b, eGFR 30–44 mL/min/1.73 m^2^; and G4, eGFR 15–29 mL/min/1.73 m^2^ [21]. Individuals with eGFR < 15 mL/min/1.73 m^2^ were excluded because this category corresponds to G5 kidney failure and may include patients receiving kidney replacement therapy, which could independently alter laboratory test results.

### 2.2. Statistical Analysis

As the extracted study data did not follow a normal distribution, all analyses were conducted using nonparametric statistical methods. Continuous variables were summarized as medians with interquartile ranges (IQR), while categorical variables were presented as percentages. Comparisons between two independent groups were performed using the Mann–Whitney U test, while the Kruskal–Wallis test was applied for analyses involving more than two groups. Spearman’s rank correlation analysis was used to examine the associations between monocyte-based inflammatory indices and renal function parameters. In addition, the diagnostic performance of monocyte-based inflammatory indices for identifying renal function abnormalities was evaluated using ROC curve analysis, with area under the curve (AUC) values reported. Risk assessment analysis was performed utilizing MedCalc Statistical Software version 20.218 (MedCalc Software Ltd., Ostend, Belgium). For regression analyses, MHR was rescaled by a factor of 100 to facilitate interpretation. Statistical analyses were performed using GraphPad Prism version 10 (GraphPad Software, San Diego, CA, USA).

## 3. Results

### 3.1. Baseline Demographic and Laboratory Characteristics of the Study Population

**Figure 1:**
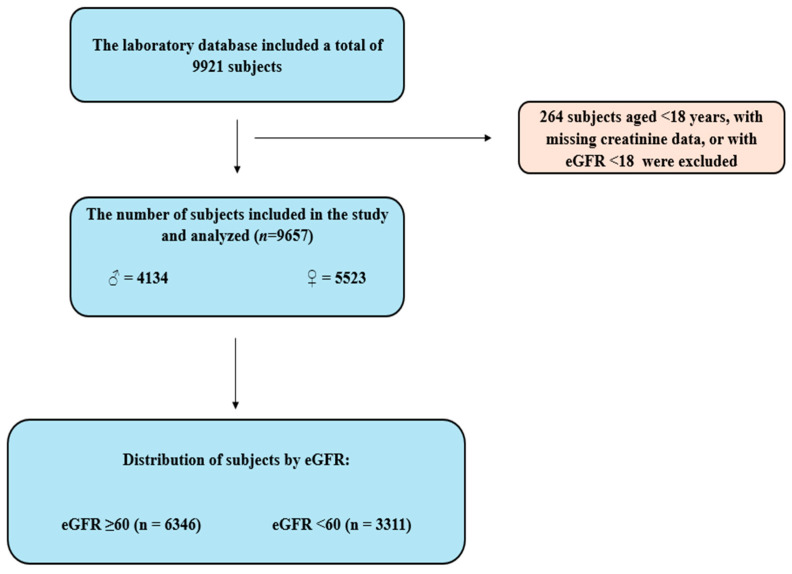
Flow chart of study population.

Laboratory and demographic data from 9921 subjects were initially identified. Of these, 9657 participants were included in the final analysis after excluding individuals younger than 18 years of age, those with missing serum creatinine data, or those with an estimated eGFR below 15 mL/min/1.73 m^2^ (Figure 1). The majority of the study population exhibited preserved renal function (eGFR ≥ 60 mL/min/1.73 m^2^), accounting for 65.71% of participants, while 34.29% had reduced eGFR (Figure 1).

As shown in Table 1, the median age of the study cohort was 37 years, with males representing 42.81% of the study population. The median eGFR was 90.34 mL/min/1.73 m^2^. Based on eGFR values, participants were classified into five categories (G1, G2, G3a, G3b, and G4).

Male participants represented 11.13%, 23.59%, and 27.84% of individuals within the G1, G2, and G3a categories, respectively. Notably, males accounted for all individuals classified within the lower renal function categories (G3b and G4), whereas no female participants were observed in these categories. In contrast, females predominated in the preserved renal function categories (G1 and G2), highlighting a distinct sex-specific distribution of renal function across eGFR categories in the Saudi general population.

### 3.2. Sex-Stratified Comparison of Monocyte-Based Inflammatory Indices According to eGFR Status

**Figure 2:**
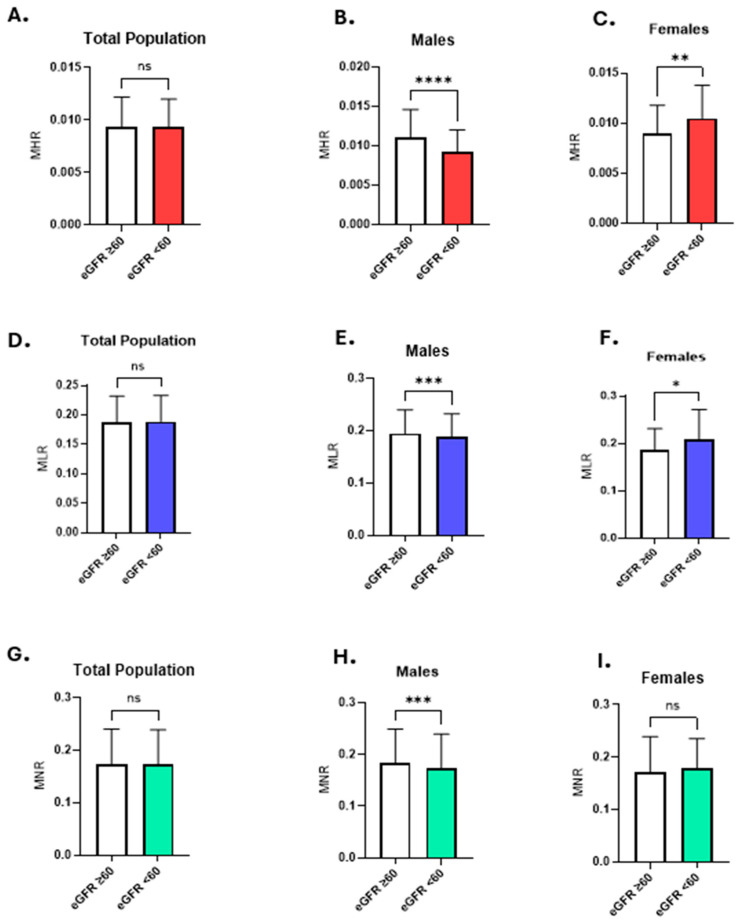
Sex-specific analysis of Monocyte-based inflammatory indices measures in relation to eGFR. A comparison of MHR patterns between preserved and reduced eGFR groups is shown for the total population (**A**), male subjects (**B**), and females (**C**). The difference in MLR measures according to eGFR status is presented for the total population (**D**), males (**E**), and females (**F**). The comparison of MNR values between subjects with preserved and reduced eGFR is presented for the total population (**G**), male participants (**H**), and females (**I**). Asterisks denote statistical significance levels: *p* < 0.05 (*), *p* < 0.01 (**), *p* < 0.001 (***), and *p* < 0.0001 (****); “ns” denotes non-significant difference.

To evaluate the association between monocyte-based inflammatory indices and renal function, the study cohort was divided according to their eGFR into preserved (≥60 mL/min/1.73 m^2^) and reduced (<60 mL/min/1.73 m^2^) eGFR groups. This cutoff was selected because an eGFR below 60 mL/min/1.73 m^2^ corresponds to KDIGO GFR category G3a or lower, reflecting at least mildly to moderately decreased kidney function [21]. In the overall population, no significant difference in MHR levels was observed between the two groups (Figure 2A). However, sex-stratified analysis revealed contrasting patterns. As shown in Figure 2B, males with reduced eGFR exhibited significantly lower MHR levels (0.0093: 0.0071–0.012) compared with those with preserved eGFR (0.011: 0.0088–0.0146). Conversely, female participants with reduced eGFR demonstrated significantly higher MHR levels (0.0105: 0.0084–0.013) than those with preserved eGFR (0.009: 0.007–0.0118) (Figure 2C).

Similarly, no significant difference in MLR levels was observed between preserved and reduced eGFR groups in the overall population (Figure 2D). In sex-stratified analyses, males with reduced eGFR showed a slightly yet significantly lower MLR levels (0.1885: 0.1538–0.2324) compared with those with preserved eGFR (0.195: 1625–0.24) (Figure 2E), whereas females with reduced eGFR exhibited significantly higher MLR levels than their counterparts with preserved eGFR (0.2075 0.1574–0.2715 vs. 0.1861: 0.1524–0.2311) (Figure 2F).

Furthermore, MNR levels did not differ significantly between preserved and reduced eGFR groups in the study cohort (Figure 2G). When stratified by sex, males with reduced eGFR showed significantly lower MNR levels compared with those with preserved eGFR (0.174: 0.1317–0.24 vs. 0.1841: 0.1432–0.25) (Figure 2H). No significant difference was observed among females, despite a trend toward higher MNR levels in individuals with reduced eGFR (Figure 2I).

### 3.3. Comparison of Monocyte-Based Inflammatory Indices Across eGFR Categories

To further investigate the relationship between monocyte-based inflammatory indices and renal function, participants were stratified into five eGFR categories (G1–G4), with G1 used as the reference group.

**Figure 3:**
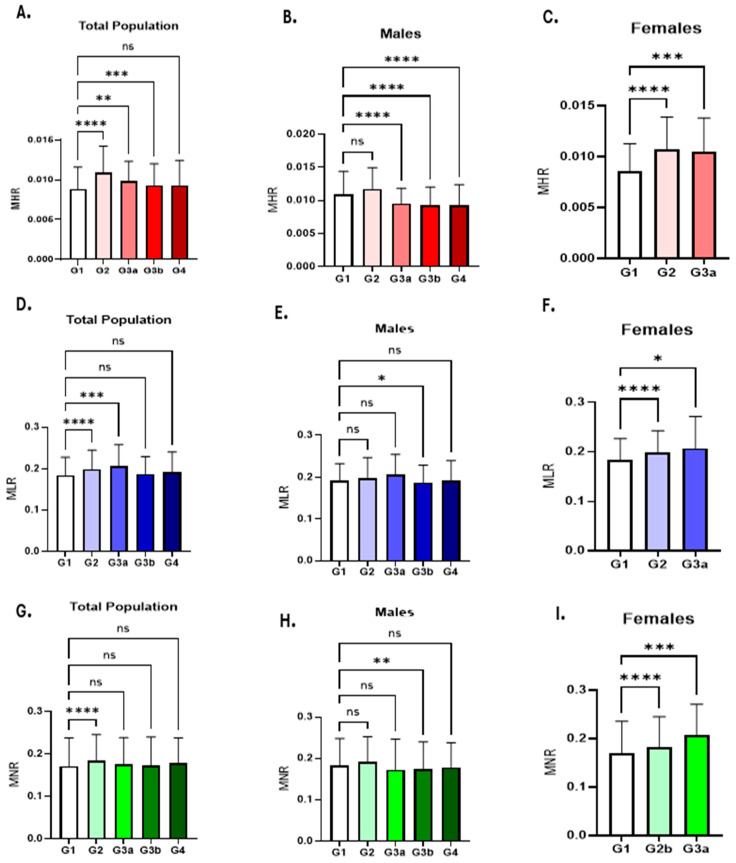
Gender-specific comparison of monocyte-based inflammatory indices across eGFR categories. The changes in MHR levels across the eGFR classes are presented for the total population (**A**), male participants (**B**), and female participants (**C**). A comparison of MLR and MNR measures across eGFR categories for the total population, male participants, and female participants are illustrated in (**D**–**F**) and (**G**–**I**), respectively. eGFR categories were defined as follows: G1, eGFR ≥90 mL/min/1.73 m^2^; G2, eGFR 60–89 mL/min/1.73 m^2^; G3a, eGFR 45–59 mL/min/1.73 m^2^; G3b, eGFR 30–44 mL/min/1.73 m^2^; and G4, eGFR 15–29 mL/min/1.73 m^2^. Advanced eGFR categories G3b and G4 included only male participants in this cohort. Asterisks denote statistical significance levels: *p* < 0.05 (*), *p* < 0.01 (**), *p* < 0.001 (***), and *p* < 0.0001 (****); “ns” denotes non-significant difference.

As shown in Figure 3A, a statistically significant difference was observed between G1 and (G2-G3b) in the total study population. In sex-stratified analyses, males exhibited an increase in MHR levels in the G2 category compared with the G1 reference group; however, this increase did not reach statistical significance. In contrast, MHR levels were significantly reduced in the G3a (0.0095: 0.007–0.0118), G3b (0.0093: 0.0071–0.012), and G4 (0.0093: 0.007–0.0124) categories compared with the G1group (0.011: 0.0086–0.0144) (Figure 3B). Female participants, on the other hand, demonstrated significantly higher MHR values in the G2 (0.0107: 0.0084–0.0139) and G3a (0.0105: 0.0084–0.0293) categories compared with those in the G1 (0.0086: 0.0067–0.01128) group (Figure 3C).

For MLR, significant differences across eGFR categories were observed in the total population, with higher MLR values in G2 and G3a compared with G1 in the total population (Figure 3D). Among males, MLR levels did not differ significantly across eGFR categories overall; however, a slight but statistically significant increase was observed in the G3a group compared with the G1 category (Figure 3E). On the other hand, a significant progressive increase in MLR measures was observed across G1–G3a classes in female subjects (Figure 3F).

Regarding MNR, the results showed a significant increase in the G2 category compared to the G1 group in the total study population, while no differences were observed across the remaining eGFR categories (Figure 3G). However, sex-specific analyses revealed divergent patterns. In males, MNR levels were slightly yet significantly lower in the G3b group compared with the G1 (Figure 3H). In females, MNR revealed a progressive elevation in MNR values in G2 and G3a groups compared with the G1 category: (G1: 0.1694 (0.127–0.2361) vs. G2: 0.182 (0.1391–0.2455) vs. G3: 0.2075 (0.1574–0.2715) (Figure 3I).

### 3.4. Association Between Monocyte-Based Inflammatory Indices and Uric Acid Levels

To further evaluate the link between monocyte-based inflammatory indices and renal function, participants were divided based on UA levels into normal UA (Norm-UA) and elevated UA (Elev-UA) groups, and the patterns of MHR, MLR, and MNR were compared between these groups.

**Figure 4:**
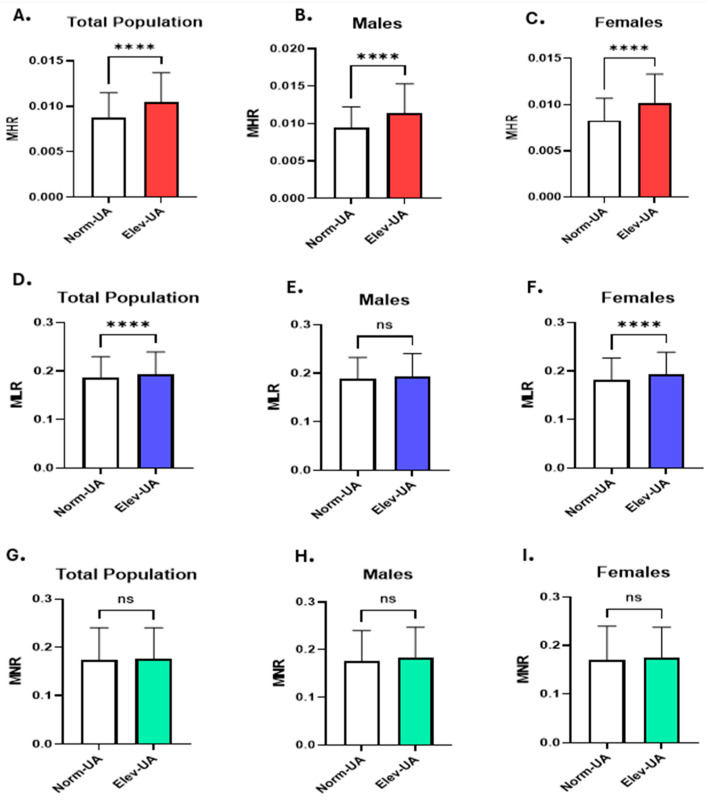
The measures of Monocyte-based inflammatory indices in relation to UA levels. A comparison of MHR patterns between normal and elevated uric acid groups is presented for the total population (**A**), males (**B**), and female subjects (**C**). The changes in MLR levels between the elevated and normal UA groups are illustrated for the total subjects (**D**), males (**E**), and females (**F**). The comparison of MNR measures between subjects with normal and high UA concentrations is shown for the total study population (**G**), male subjects (**H**), and females (**I**). Asterisks mark the significance level; **** for *p* < 0.0001, and “ns” denotes a non-significant difference.

As shown in Figure 4A, individuals in the Elev-UA group exhibited significantly higher MHR levels (0.0105: 0.0081–0.0137) compared with those in the Norm-UA group (0.0088: 0.0069–0.0088) (Figure 4A). This pattern remained consistent when both genders were analyzed separately (Figure 4B,C).

MLR showed a slight, yet statistically significant increase in the Elev-UA group compared with the Norm-UA group in the total study population (Figure 4D). Sex-stratified analysis showed similar results when females were solely analyzed (Elev; 0.1923: 0.155–0.2388 vs. Norm; 0.1826: 0.1513–0.2268), but not in male subjects (Figure 4E,F).

In contrast, no significant differences in MNR levels were detected between the Norm-UA and Elev-UA groups in the total population or when analyses were stratified by sex (Figure 4 G–I).

### 3.5. Comparison of Monocyte-Based Inflammatory Across UA Tertiles

**Figure 5:**
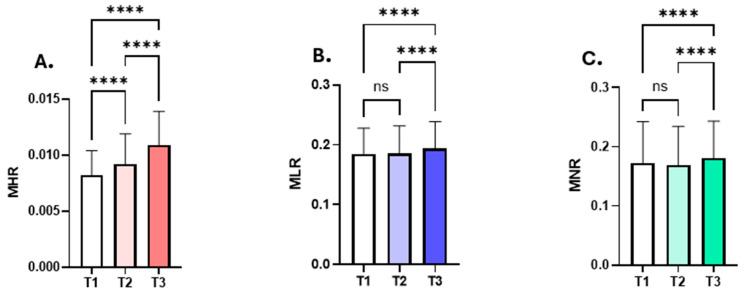
Comparison of Monocyte-based inflammatory ratios across UA tertiles. Subjects were categorized into three tertiles based on their UA values (T1 = lowest, T2 = middle, T3 = highest). The differences in MHR, MLR, and MNR levels between the study tertiles are shown in (**A**–**C**), respectively. Asterisks mark the significance level; **** for *p* < 0.0001, and “ns” denotes a non-significant difference.

To further examine the association between monocyte-based inflammatory indices and UA levels, subjects were stratified into three tertiles based on their UA concentrations. The analysis showed a clear, significant progressive increase in MHR levels across increasing UA tertiles (MHR; 0.0082: 0.0063–0.0104 vs. 0.0092: 0.0071–0.012 vs. 0.011: 0.0084–0.014) (Figure 5A). In comparison, both MLR and MNR demonstrated a slight yet statistically significant elevation in the T3 compared with T1 and T2, with no significant difference observed between T1 and T2 (Figure 5B,C).

### 3.6. Correlation of MHR, MLR, and MNR with Renal Function Indices in the Study Population

A Spearman correlation analysis was performed to further examine the associations between monocyte-based inflammatory indices and renal function parameters in the study population. MHR demonstrated the largest correlation coefficients with eGFR (males: ρ = 0.142, *p* < 0.0001; females: ρ = −0.18, *p* < 0.0001) and UA (males: ρ = 0.321, *p* < 0.0001; females: ρ = 0.277, *p* < 0.0001) compared with MLR and MNR in both sexes (Table 2).

### 3.7. Risk Assessment of Monocyte-Based Inflammatory Indices in Relation to Renal Function Abnormalities

Risk assessment analyses were performed to evaluate the associations between elevated monocyte-based inflammatory indices and renal function, with analyses stratified by sex (Table 3).

Among female participants, elevated MHR and MLR were significantly associated with a higher risk of reduced eGFR (MHR: PR = 1.38, 95% CI: 1.15–1.66, *p* = 0.0005; MLR: PR = 1.24, 95% CI: 1.01–1.52, *p* = 0.0357). Consistently, females with elevated MHR and MLR demonstrated significantly increased odds of reduced eGFR (OR = 2.09, 95% CI: 1.24–3.52, *p* = 0.0054; OR = 1.609, 95% CI: 0.968–2.67, *p* = 0.0661). No significant association was observed between MNR and reduced eGFR in female subjects.

In contrast, elevated MHR was inversely associated with reduced eGFR (PR = 0.71, 95% CI: 0.67–0.748, *p* < 0.0001; OR = 0.415, 95% CI: 0.35–0.487, *p* < 0.0001) among males. Similarly, elevated MLR and MNR were associated with a lower risk of having reduced eGFR in males (MLR: PR = 0.91, 95% CI: 0.8524–0.9780, *p* = 0.0095; MNR: PR = 0.903, 95% CI: 0.843–0.967, *p* = 0.0035); and decreased chance of having reduced eGFR (MLR: OR = 0.825, 95% CI: 0.711–0.958, *p* = 0.0117; MNR: OR = 0.806, 95% CI: 0.694–0.935, *p* = 0.0047).

Furthermore, Prevalence ratios showed the strongest associations of MHR with hyperuricemia for both genders (Males: PR = 1.38, 95% CI: 1.3064–1.4730, *p* < 0.0001; Females: PR = 1.55, 95% CI: 1.4705–1.6412, *p* < 0.0001). Odds ratios demonstrated similar patterns in males and females (Males: OR = 2.383, 95% CI: 1.9636–2.8920, *p* < 0.0001; Females: OR = 2.3811, 95% CI: 2.1332–2.6578, *p* < 0.0001). The results showed increased MLR levels were associated with higher risks of hyperuricemia in female subjects but not males (PR = 1.136, 95% CI: 1.0765–1.1994, *p* < 0.0001), and 1.28 times higher the chance of having hyperuricemia. No association was observed between MNR and hyperuricemia in both genders.

### 3.8. Diagnostic Performance of Monocyte-Based Inflammatory Indices for Renal Function Abnormalities

To evaluate the discriminatory performance of monocyte-based inflammatory indices for identifying renal function abnormalities in the study cohort, ROC curve analysis was performed. Although the overall discrimination was modest, MHR consistently demonstrated better discriminatory performance than MLR and MNR in both genders (Table 4). MHR exhibited better discriminatory ability for identifying subjects with reduced eGFR in males (AUC = 0.646, *p* < 0.0001) and females (AUC = 0.610, *p* = 0.0031) than MLR and MNR. Similarly, MHR showed the highest diagnostic accuracy for hyperuricemia in both sexes, compared with MLR and MNR.

### 3.9. Regression Analysis of Monocyte-Based Inflammatory Indices and eGFR

The regression analysis supported the distinct associations between monocyte-based inflammatory indices and eGFR in the study population (Table 5). In males, the analysis showed a significant positive association between MHR and eGFR, such that each 0.01 increase in MHR was associated with a 9.90 mL/min/1.73 m^2^ increase in eGFR (*p* < 0.0001). In contrast, MHR showed a significant inverse association with eGFR, as each 0.01 increase in MHR was associated with a 9.416 mL/min/1.73 m^2^ decrease in eGFR (*p* < 0.0001) in female subjects. These associations remained significant after adjustment for the other laboratory parameters. MLR showed a similar but weaker gender-divergent pattern, while MNR was not significantly associated with eGFR after adjustment in either sex.

## 4. Discussion

This study highlights the potential value of CBC-derived monocyte-based inflammatory indices as accessible markers of kidney dysfunction in the general adult population. Because these indices are inexpensive, routinely available, and easily calculated from standard laboratory testing, they offer practical translational relevance compared with specialized inflammatory biomarkers that are less feasible in everyday clinical settings [24,25,26,27]. In the context of CKD, where persistent low-grade inflammation contributes to renal injury, endothelial dysfunction, and disease progression, such markers may provide a useful framework for identifying individuals at increased risk of renal impairment [28,29]. Importantly, MHR should not be viewed as a replacement for established kidney function markers such as serum creatinine or eGFR. Rather, because it combines monocyte count with HDL-C, both of which are commonly measured as part of routine hematological and lipid profiles, MHR may provide additional inflammatory-metabolic information that complements conventional renal assessment. This may be particularly useful for early risk stratification and population-level screening approaches in settings where routine laboratory data are already available. This may be particularly useful for early risk stratification and population-level assessment in individuals undergoing routine health screening, including those with available CBC and lipid profile results but no specific kidney-focused evaluation.

The main finding of the present study is that MHR emerged as the strongest and most consistent inflammatory index associated with renal function among the evaluated monocyte-based markers. MHR showed the strongest correlations with eGFR and UA, the best discriminatory performance for reduced eGFR and hyperuricemia, and remained independently associated with lower eGFR in regression analysis. This likely reflects its greater biological relevance, as MHR captures both monocyte-driven inflammatory activation and reduced HDL-mediated anti-inflammatory and antioxidant protection. In contrast to MLR and MNR, which reflect leukocyte balance alone, MHR may better represent the broader inflammatory-metabolic disturbances that accompany declining kidney function. This interpretation is further supported by recent evidence demonstrating the prognostic significance of MHR in CKD. In a large analysis of older adults with CKD derived from the National Health and Nutrition Examination Survey (NHANES) 1999–2018, elevated MHR independently predicted both all-cause and renal-specific mortality even after adjustment for eGFR, albuminuria, and major clinical covariates [30]. Importantly, MHR was identified alongside eGFR and albumin-to-creatinine ratio as one of the strongest predictors of renal-specific mortality, underscoring its value beyond traditional renal markers. Additional support comes from a retrospective study in patients with type 2 diabetes, in which MHR was significantly associated with diabetic kidney disease and increased progressively with worsening albuminuria [31]. In that study, higher MHR independently predicted DKD risk in multivariable analysis, further supporting its relevance as a marker of renal injury and progression. While these studies examined different renal outcomes in distinct clinical populations, and the present study evaluated inflammatory associations across renal function status in a broader adult population, the consistent signal across these settings strengthens the view that MHR is not merely an inflammatory correlate, but a clinically meaningful marker of renal risk.

A second major finding is the clear sex-specific divergence in these associations. In females, reduced eGFR was associated with higher MHR and MLR, and elevated levels of these indices were linked to a greater risk of renal dysfunction. In males, however, reduced eGFR was associated with lower MHR, MLR, and MNR, with inverse risk associations observed for these markers. These contrasting patterns suggest that the inflammatory profile accompanying renal impairment is not biologically uniform between the sexes. Sex-related differences in kidney disease may be influenced by the combined effects of sex chromosomes, sex hormones, renal structure, kidney hemodynamics, vascular function, lipid metabolism, and immune-inflammatory regulation. Estradiol has been suggested to exert renoprotective effects through favorable modulation of endothelial function, renal blood flow, inflammatory signaling, oxidative stress, and fibrotic pathways, whereas testosterone may promote glomerular hyperfiltration, vasoconstrictive responses, oxidative stress, and profibrotic mechanisms [8]. In addition, sex hormones can regulate immune-cell activity and inflammatory gene expression, which may contribute to sex-dependent differences in circulating leukocyte profiles and inflammatory biomarker behavior [8,32,33]. These mechanisms may partly explain why the same monocyte-based indices showed opposite directions of association in males and females in the present study. Therefore, sex is not merely a confounder but an important biological modifier of biomarker performance in kidney disease [8,32,33].

An important consideration in the current study is that the more advanced eGFR categories, particularly G3b and G4, were composed exclusively of male participants, with no female representation in these strata. While this imbalance may partly influence comparisons at advanced stages of renal dysfunction, it does not fully explain the observed sex-specific differences. Notably, divergent trends were already apparent in earlier eGFR categories shared by both sexes. MHR was reduced in males at G3a but increased in females, while MLR and MNR tended to rise in females from G2 onward without comparable changes in males. Thus, the persistence of these differences in overlapping eGFR categories supports the interpretation that the observed divergence reflects genuine biological variation rather than only sample distribution.

The UA findings further strengthen the clinical relevance of MHR. In the present study, MHR increased consistently in hyperuricemia and across rising UA tertiles, reinforcing its role as a marker of renal-metabolic inflammatory burden. This is supported by large population-based evidence from NHANES showing that higher MHR is independently associated with hyperuricemia prevalence, with an even stronger predictive signal observed in females [34]. Consistent findings have also been reported in type 2 diabetes mellitus, where elevated MHR independently predicted hyperuricemia risk, further underscoring the link between MHR, metabolic dysfunction, and systemic inflammation [35]. Likewise, a study in Chinese adults found that MHR was significantly and positively correlated with serum uric acid levels even after adjustment for multiple clinical and lifestyle-related confounders, supporting the reproducibility of this association across different populations [36]. Furthermore, a study conducted by Mi et al., including 7247 participants, showed that higher MHR is also associated with increased gout risk, with an even stronger relationship observed among gout patients with renal dysfunction, suggesting that MHR may reflect not only UA-related inflammatory burden but also the severity of renal involvement in urate-associated disease [37]. Collectively, these findings position MHR as the most clinically promising index among the evaluated markers in the current study. Its simplicity, availability, and reproducible association with both renal dysfunction and hyperuricemia suggest that it may serve as a useful adjunctive marker for sex-informed risk stratification.

The present study has several strengths. First, the large sample size and the use of routinely collected laboratory data, supported by automated data acquisition, enhance the accuracy and reliability of the measurements. Second, the relatively homogeneous study population reduces potential confounding related to ethnic variability. Finally, the inclusion of individuals from the general population, rather than selected clinical cohorts, minimizes selection bias and improves the broader applicability of the findings. However, some limitations should be considered. The retrospective design precludes causal inference, and the available dataset did not include detailed information on comorbidities or medication use, which may influence kidney function and inflammatory status. Future studies incorporating these variables along with anthropometric measures would allow more comprehensive adjustment for potential confounders and strengthen the interpretation of these associations. In addition, advanced renal dysfunction categories, specifically G3b and G4, included only male participants. This unequal sex distribution may influence the interpretation of sex-specific differences and limit the generalizability of findings related to advanced eGFR categories. Therefore, these results should be interpreted with caution. Prospective studies with more comprehensive clinical characterization are needed to validate these findings and clarify the mechanisms underlying the observed sex-specific associations.

## 5. Conclusions

In conclusion, this study demonstrates that monocyte-based inflammatory indices are associated with renal dysfunction and hyperuricemia in a sex-dependent manner, in a general adult population, with MHR showing the strongest and most consistent performance across analyses. These findings support the potential utility of MHR as a simple, readily available adjunctive biomarker that may enhance sex-informed risk assessment and early risk stratification in individuals undergoing routine laboratory evaluation. However, due to the retrospective cross-sectional design of the current study, the present findings should be interpreted as associative rather than causal. Given the predominantly preserved renal function of the study population, further prospective and mechanistic studies including individuals with clinically significant renal impairment are needed to confirm its clinical applicability and define its role in personalized renal risk stratification.

## Figures and Tables

**Table 1:** The demographic and laboratory baseline characteristics of the study population.

Demographic Variables	Median (±IQR)
Age (Years)	37 (32–46)
Sex male (%)	4134 (42.81%)
eGFR (mL/min/1.73 m^2^)	90.34 (38.38–113.6)
eGFR categories	
G1 male (%)	546 (11.13%)
G2 male (%)	340 (23.59%)
G3a male (%)	169 (27.84%)
G3b male (%)	2673 (100%)
G4 male (%)	406 (100%)
Lab parameters	
AST (U/L)	20 (16–25)
monocyte (×10^9^/L)	0.44 (0.36–0.54)
TSH (mIU/L)	1.70 (1.17–2.49)
Creatinine (mg/dL)	0.7 (0.60–0.86)
FBG (mg/dL)	95 (87–108)
Total cholesterol (mg/dL)	198 (169–224)
UA (mg/dL)	5.2 (4.2–6.4)

Abbreviations: eGFR, estimated glomerular filtration rate; AST, aspartate aminotransferase; TSH, thyroid-stimulating hormone; FBG, fasting blood glucose; UA, uric acid. eGFR categories were defined according to KDIGO GFR categories as follows: G1, eGFR ≥ 90 mL/min/1.73 m^2^; G2, eGFR 60–89 mL/min/1.73 m^2^; G3a, eGFR 45–59 mL/min/1.73 m^2^; G3b, eGFR 30–44 mL/min/1.73 m^2^; and G4, eGFR 15–29 mL/min/1.73 m^2^.

**Table 2:** Correlation of monocyte-based inflammatory markers with renal function indices in the study population.

Parameter	Index	ρ	*p*-Value
eGFR	Males		
	MHR	0.142	<0.0001
	MLR	0.042	0.007
	MNR	0.009	0.57
eGFR	Females		
	MHR	−0.180	<0.0001
	MLR	−0.059	<0.0001
	MNR	−0.043	0.0014
UA	Males		
	MHR	0.321	<0.0001
	MLR	0.046	0.003
	MNR	0.050	0.0015
UA	Females		
	MHR	0.277	<0.0001
	MLR	0.0481	0.0004
	MNR	0.01322	0.3264

Abbreviations: eGFR, estimated glomerular filtration rate; UA, uric acid; MHR, monocyte-to-HDL cholesterol ratio; MLR, monocyte-to-lymphocyte ratio; and MNR, monocyte-to-neutrophil ratio; ρ, Spearman’s rank correlation coefficient.

**Table 3:** Risk assessment analysis of monocyte-based inflammatory indices in relation to renal function parameters.

eGFR						UA			
Index		Score	95% CI	Z Statistic	*p*-Value	Score	95% CI	Z Statistic	*p*-Value
	PR								
	Males	0.7093	0.6718–0.7489	12.392	*p* < 0.0001	1.3872	1.3064–1.4730	10.69	*p* < 0.0001
	Females	1.3826	1.1513–1.6604	3.469	*p* = 0.0005	1.5535	1.4705–1.6412	15.724	*p* < 0.0001
MHR	OR								
	Males	0.4154	0.3538–0.4877	10.733	*p* < 0.0001	2.383	1.9636–2.8920	8.792	*p* < 0.0001
	Females	2.0957	1.2450–3.5275	2.785	*p* = 0.0054	2.3811	2.1332–2.6578	15.467	*p* < 0.0001
	PR								
	Males	0.913	0.8524–0.9780	2.595	*p* = 0.0095	1.0698	0.9859–1.1610	1.619	*p* = 0.1055
	Females	1.2418	1.0145–1.5200	2.1	*p* = 0.0357	1.1363	1.0765–1.1994	4.635	*p* < 0.0001
MLR	OR								
	Males	0.8254	0.7110–0.9581	2.522	*p* = 0.0117	1.1527	0.9659–1.3756	1.576	*p* = 0.1151
	Females	1.6094	0.9688–2.6735	1.838	*p* = 0.0661	1.2868	1.1555 to 1.4331	4.591	*p* < 0.0001
	PR								
	Males	0.903	0.8432–0.9670	2.921	*p* = 0.0035	1.0624	0.9785–1.1534	1.443	*p* = 0.1491
	Females	1.1335	0.9074–1.4160	1.104	*p* = 0.2696	1.0367	0.9819–1.0946	1.3	*p* = 0.1937
MNR	OR								
	Males	0.8062	0.6944 to 0.9359	2.83	*p* = 0.0047	1.1354	0.9515–1.3548	1.408	*p* = 0.1590
	Females	1.3004	0.7889–2.1436	1.03	*p* = 0.3029	1.0736	0.9642–1.1955	1.296	*p* = 0.1951

Abbreviations: eGFR, estimated glomerular filtration rate; UA, uric acid; MHR, monocyte-to-HDL cholesterol ratio; MLR, monocyte-to-lymphocyte ratio; and MNR, monocyte-to-neutrophil ratio; CI, confidence interval.

**Table 4:** Discriminatory performance of monocyte-based inflammatory ratios for identifying reduced eGFR and hyperuricemia.

Parameter	Indices	AUC	95% CI	*p*-Value
	Males			
eGFR	MHR	0.646	0.6265–0.6662	*p* < 0.0001
	MLR	0.537	0.5166–0.5589	*p* = 0.0006
	MNR	0.542	0.5211–0.5625	*p* = 0.0001
	Females			
eGFR	MHR	0.61	0.5386–0.6779	*p* = 0.0031
	MLR	0.586	0.5109–0.6610	*p* = 0.0188
	MNR	0.545	0.4827–0.6076	*p* = 0.2169
	Males			
UA	MHR	0.65	0.6248–0.6729	*p* < 0.0001
	MLR	0.522	0.4973–0.5484	*p* = 0.0787
	MNR	0.52	0.4958–0.5458	*p* = 0.1089
	Females			
UA	MHR	0.642	0.6271–0.6567	*p* < 0.0001
	MLR	0.535	0.5195–0.5506	*p* < 0.0001
	MNR	0.513	0.4978–0.5286	*p* = 0.094

Abbreviations: eGFR, estimated glomerular filtration rate; UA, uric acid; MHR, monocyte-to-HDL cholesterol ratio; MLR, monocyte-to-lymphocyte ratio; and MNR, monocyte-to-neutrophil ratio; CI, confidence interval; AUC, area under curve.

**Table 5:** Multivariable linear regression analysis of the association between monocyte-based inflammatory indices and eGFR.

		Model 1			Model 2		
Gender	Indices	β Estimate	(95% CI)	*p*-Value	β Estimate	(95% CI)	*p*-Value
Males	MHR	9.902	8.353 to 11.45	<0.0001	9.422	7.881 to 10.96	<0.0001
	MLR	0.14	0.047 to 0.232	0.003	0.136	0.044 to 0.227	0.0036
	MNR	0.062	−0.008 to 0.132	0.0832	0.029	−0.041 to 0.099	0.417
Females	MHR	−9.416	−10.54 to −8.29	<0.0001	−9.224	−10.34 to −8.11	<0.0001
	MLR	−0.117	−0.175 to −0.059	<0.0001	−0.109	−0.166 to −0.052	0.0002
	MNR	−0.052	−0.102 to −0.001	0.047	−0.039	0–0.089 to 0.012	0.1329

Model 1: unadjusted analysis; Model 2: adjusted for FBG, AST, TSH, and total cholesterol. Abbreviations: β, regression coefficient; 95% CI, 95% confidence interval; MHR, monocyte-to-HDL cholesterol ratio; MLR, monocyte-to-lymphocyte ratio; MNR, monocyte-to-neutrophil ratio.

## Data Availability

The datasets generated and/or analyzed during the current study are not publicly available due to institutional restrictions. Access may be granted upon reasonable request to the corresponding author, subject to approval by the relevant data-providing institution.
